# Supplementary figures and images for: Expanding the fluorescent toolkit: Blue fluorescent protein-expressing Plasmodium berghei for enhanced multiplex microscopy
Source: PLoS One. 2025 Mar 3;20(3):e0308055. doi: 10.1371/journal.pone.0308055 (PMC11875362; doi:10.1371/journal.pone.0308055)

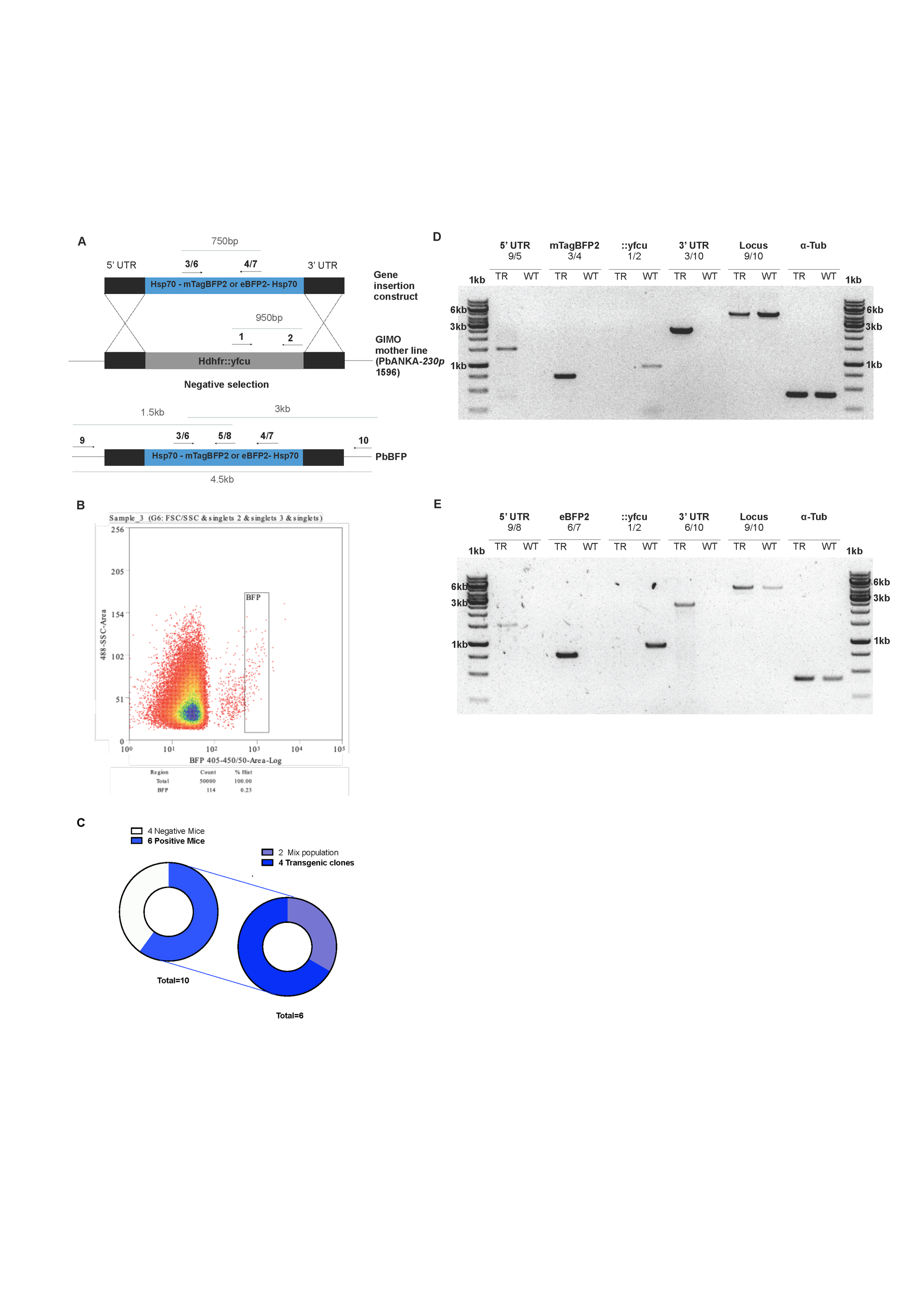

Supplement: S1 Fig — (A) showing the GIMO mother line PBANKA 230p 1596 locus, with the 3UTR and the 5’ untranslated region (5’ UTR) respectively flanked by the selection marker hdhfr::yfcu (black boxes). mTagBFP2 and eBFP2 were substituted for the yfcu selectable marker by homologous recombination under the Hsp70 promoter. Primers designed for the integration of PCR were indicated in the illustration and listed in Table 2. (B) Flow cytometry profile of the PbmTagBFP2 parasite when the parasitemia reached 0.23%. (C) Venn diagram displaying the limiting dilution of the PbeBFP2 parasite lines where 6 out of 10 mice tested positive. Integration PCR allowed for the identification of four of the six transgenic parasites that had no wild type. (D) Integration PCR results confirmed the proper integration of the mTagBFP2 and eBFP2 constructs. The expected size of the BFP fragment was determined to be 735 bp (solely in the TR), the yfcu fragment was 950 bp (exclusive to the WT), the 5’ UTR (solely in the TR) at 1.5 kilobases, the 3’ UTR (solely in the TR) at 3 kilobases, and the complete locus construct at 4.5kb. α-Tubulin was used as a control. TR = transgenics; WT = wild type, GIMO mother line (1596cl1); α-Tub = α-Tubulin. (TIF) [file pone.0308055.s001.tif]

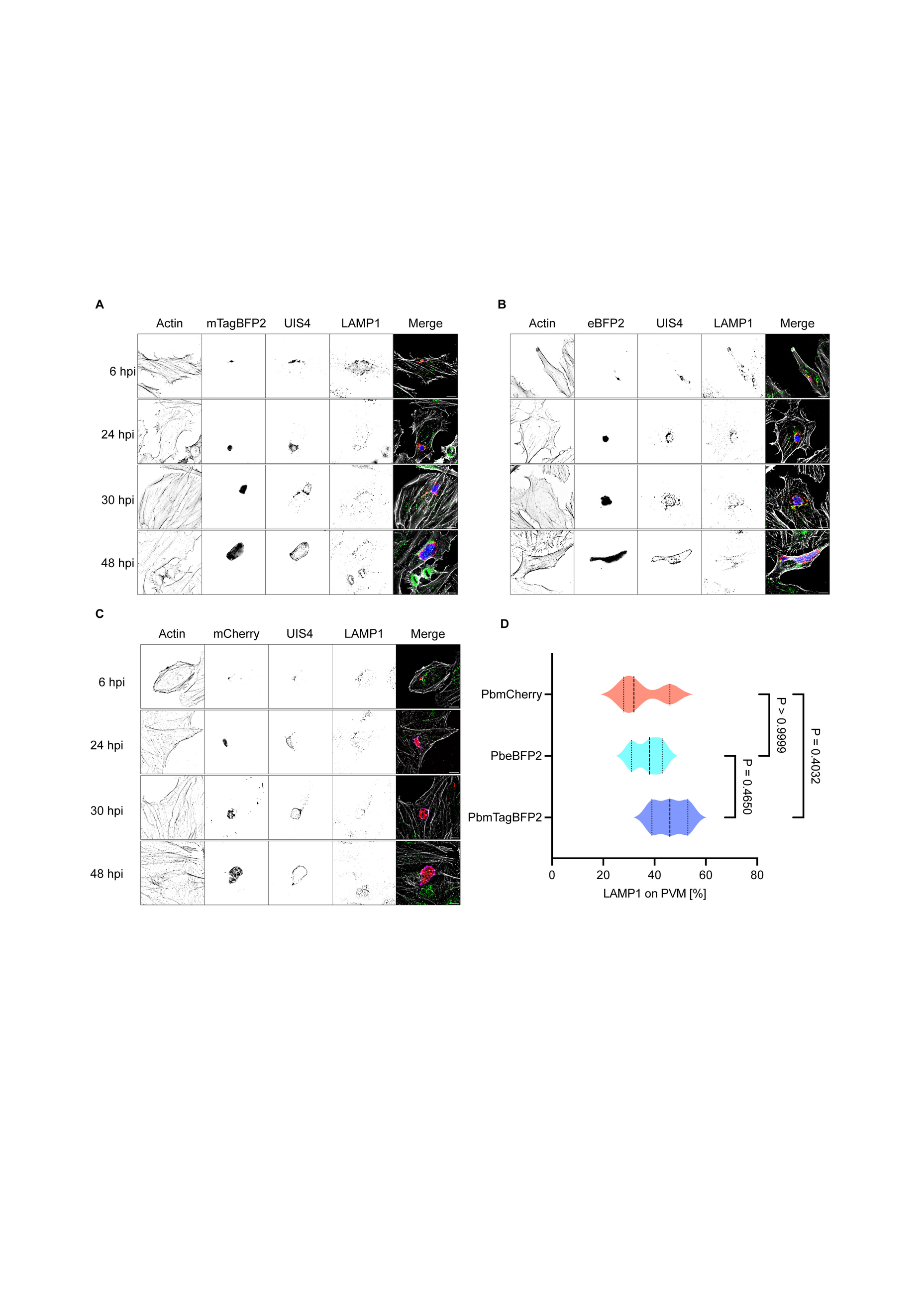

Supplement: S2 Fig — (A), (B), and (C) immunofluorescent assays of fixed infected HeLa cells showing the host-cell lysosome vesicles interaction with the parasite PVM. HeLa cells were infected with sporozoites of the respective parasite lines and fixed at various time points (6, 24, 30, 48, and 56 hpi) during parasite development. Anti-UIS4 was used to stain the parasite PVM (magenta), anti-LAMP1 was used to stain the host cell lysosome (green), and phalloidin dye conjugated to Alexa Fluor 488 was used to stain the host cell polymerized actin (grey). The cells were imaged using a Nikon Crest V3 microscope in widefield mode. Z-stack images of the whole cell were acquired, and the host lysosome interaction with the parasite PVM was analyzed using the 3D and 4D image analysis software Imaris as described in the methods section. (D) Violin plot showing the percentage of host lysosomes (stained with anti-LAMP1) attached (0 μm) up to close proximity (0.5 μm) of the parasite PVM at 24 hpi. Neither of the BFP transgenic parasites differed significantly from the PbmCherry, with an average of 32%, 39%, and 46%, respectively, of PbmCherry, PbeBFP2, and PbmTagBFP2 LAMP1, present in the vicinity of the parasite PVM. Twenty infected cells from each transgenic parasite were analyzed in triplicate. The data were structured and analyzed using GraphPad Prism version 9.0.0 for Mac, GraphPad Software, San Diego, California, USA, www.graphpad.com. The statistical comparison tests were carried out using one-way ANOVA with Tukey’s multiple comparisons test (*p ≤ 0.05, **p ≤ 0.01, ***p ≤ 0.001). The images were deconvolved with Huygens Professional version [Huygens Remote Manager v3.8] (Scientific Volume Imaging, The Netherlands, http://svi.nl). Scale bars = 10 μm. (TIF) [file pone.0308055.s002.tif]
