# Supplementary material for: Expanding the fluorescent toolkit: Blue fluorescent protein-expressing Plasmodium berghei for enhanced multiplex microscopy
Source: PLoS One. 2025 Mar 3;20(3):e0308055. doi: 10.1371/journal.pone.0308055 (PMC11875362; doi:10.1371/journal.pone.0308055)

S1 Fig D agarose gel image. Gel imaged with U:Genus 3, SYNGENE, in invert mode (black and white)

Tr = transgenic, WT = wild-type; referer to S1_Fig.

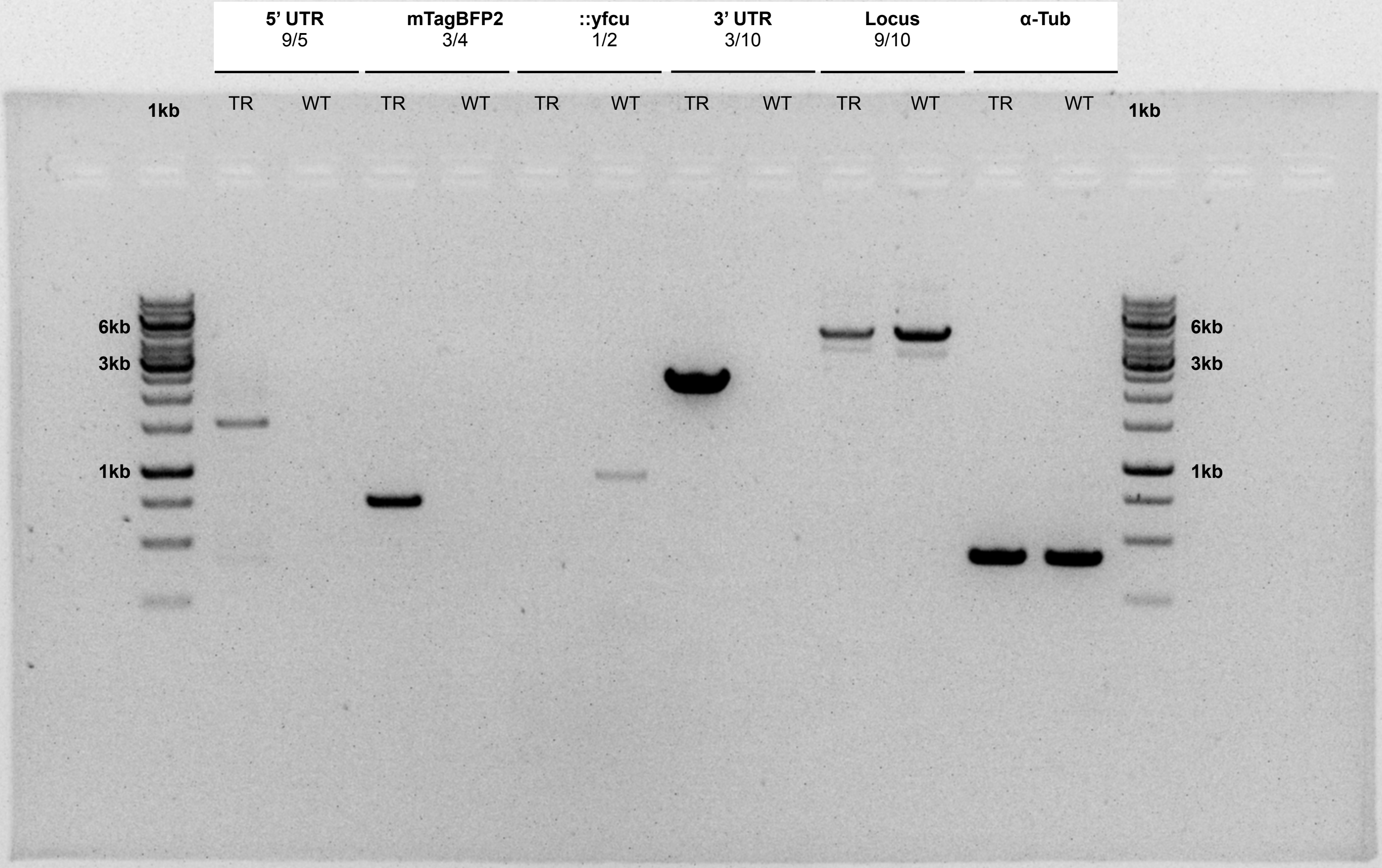

S1 Fig E agarose gel image. Gel imaged with U:Genus 3, SYNGENE, in invert mode (black and white)

Tr = transgenic, WT = wild-type; referer to S1_Fig.

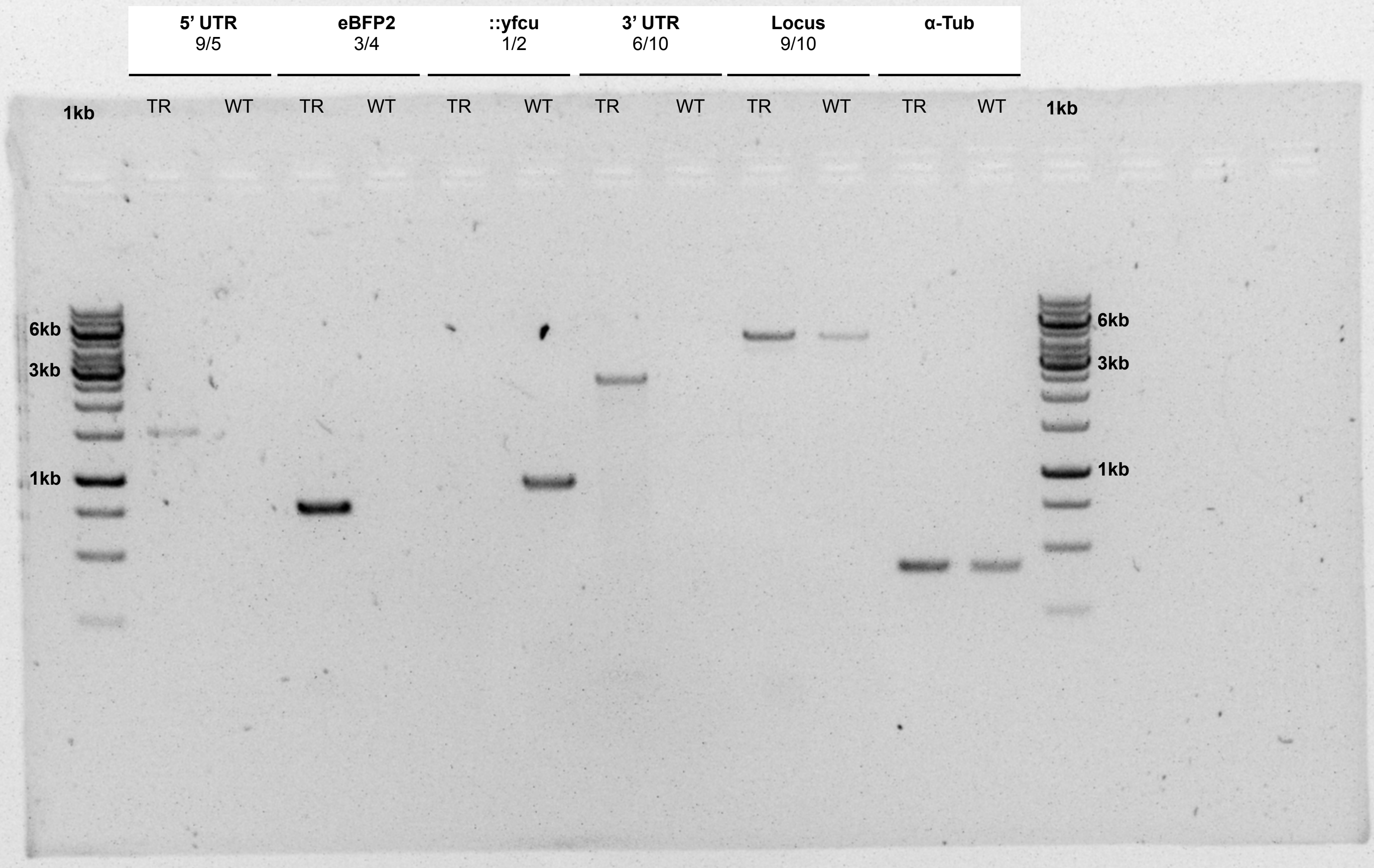

Supplement: S1 Raw images — (PDF) [file pone.0308055.s011.pdf]
